# Health systems research in Lao PDR: capacity development for getting research into policy and practice

**DOI:** 10.1186/1478-4505-5-11

**Published:** 2007-10-16

**Authors:** Kristina Jönsson, Göran Tomson, Christer Jönsson, Sengchanh Kounnavong, Rolf Wahlström

**Affiliations:** 1 Centre for East and South-East Asian Studies, Lund University, Scheelevägen 15, SE-223 70 Lund, Sweden; 2 Division of International Health (IHCAR), Department of Public Health Sciences, Karolinska Institutet, SE-171 77 Stockholm, Sweden; 3 Medical Management Centre (MMC), Karolinska Institutet, SE-171 77 Stockholm, Sweden; 4 Department of Political Science, Lund University, Box 52, SE-221 00 Lund, Sweden; 5 National Institute of Public Health (NIOPH), Ministry of Health, Vientiane, Lao PDR

## Abstract

**Background:**

Lao PDR is a low-income country with an urgent need for evidence-informed policymaking in the healthcare sector. During the last decade a number of Health Systems Research (HSR) projects have been conducted in order to meet this need. However, although knowledge about research is increasing among policymakers, the use of research in policymaking is still limited.

**Methods:**

This article investigates the relationship between research and policymaking from the perspective of those participating in HSR projects. The study is based on 28 interviews, two group discussions and the responses from 56 questionnaires.

**Results:**

The interviewees and questionnaire respondents were aware of the barriers to getting research into policy and practice. But while some were optimistic, claiming that there had been a change of attitudes among policymakers in the last two years, others were more pessimistic and did not expect any real changes until years from now. The major barriers to feeding research results into policy and practice included an inability to influence the policy process and to get policymakers and practitioners interested in research results. Another barrier was the lack of continuous capacity development and high-quality research, both of which are related to funding and international support. Many of the interviewees and questionnaire respondents also pointed out that communication between those conducting research and policymakers must be improved.

**Conclusion:**

The results show that in the case of Lao PDR, research capacity development is at a crucial stage for implementing research into policy and practice. If research is going to make a consistent impact on policymaking in the Lao health care sector, the attitude towards research will need to be changed in order to get research prioritised, both among those conducting research, and among policymakers and practitioners. Our findings indicate that there is awareness about the barriers in this process.

## Introduction

This study seeks to contribute to the current discussion about health research feeding into policymaking in a low-income country setting. It is a follow-up of a previous article about research and policymaking in Lao PDR focusing on decision-makers and the usefulness of research evidence in policy implementation. It was concluded that health officials were very positive towards research, but that few seemed to fully understand what it entails, which underlines an importance "to focus on how research is understood and communicated in order to ensure successful implementation of policies [1]." In order to further explore facilitating factors and obstacles in the process of implementing research into policy and practice, this article takes a somewhat less explored viewpoint, namely the perspective of those participating in Health System Research (HSR) projects [cf. [2]]. Accordingly, the study focuses on perceptions about how findings from HSR projects are or should be communicated and used for decision-making together with capacity development of the participants. In the case of Lao PDR, capacity development is a necessary first step towards linking research to policymaking.

## Background

The last two decades have seen a growing need for health sector reforms in many Low- and Middle Income Countries (LMIC), due primarily to unsatisfactory quality of heath care services. Conversely, a parallel discussion about how to implement research into policy and practice in order to support adequate policymaking has flourished [3]. A large number of publications can be found in the field, but most of them emanate from countries belonging to the Organisation for Economic Co-operation and Development. Recently, the Overseas Development Institute produced a number of working papers and articles focusing on international development that deal specifically with research and policymaking in LMIC [4]. Additionally, in 2004, the World Health Organisation (WHO) organised the Ministerial Summit on Health Research where health research was emphasised as a role in meeting the Millennium Development Goals [5]. Despite these efforts to acknowledge the neglect of research in policymaking, since 1995 only a few reports have addressed this issue [1,2,6-10].

The reason why research and policymaking to a large extent lead separate lives has been explained by the different worldviews of researchers and policymakers [11-13]. Often research is only one source of information among many for policymakers, and consequently, the contribution of researcher may be neglected. Moreover, research results are not always clearly presented, making the information inaccessible. Other obstacles are the researchers' lack of knowledge of the policymaking process, stakeholders' lack of ownership of the research agenda, and inappropriate institutional framework linking stakeholders and researchers [3,14,15]. Yet another conclusion to be drawn from the literature is that research primarily has an "enlightening function" in policymaking, rather than actually steering the decision-making process [16], i.e. the interaction between researchers and policymakers is thought only to influence the understanding of and intent for use of research, but is not directly impacting policy formulation and implementation [9,17]. Adding to this complexity is the fact that policymaking is rarely a linear and rational process where problems are identified and followed by informed decisions that are later implemented and evaluated [18,19]. Often there is a need for a "window of opportunity" for research to have an impact, and the timing of when that is going to happen is very difficult to predict for researchers as well as for policymakers [15,20].

The use of analytical policy frameworks derived from European and North American settings, which assume policy processes and feed-back mechanisms based on principles common in more developed democracies, has bearing on policy analysis and the conclusions drawn from studies made in LMIC. Frequently the political and bureaucratic structures are different, as is the political culture. There are also no independent think tanks, social movements, or sources providing profound academic research that may influence the policy agenda, something that is taken for granted in more developed countries. Instead, donors play an important role in setting the policy agenda or assisting in the implementation phase [21]. However, external influence in the policy process can be sensitive, as it may question existing power hierarchies [17,22], and because external actors become involved in domestic political life [18]. Hence, more studies from LMIC settings are warranted in order to get a comprehensive picture of the relationship between research and policymaking, and the role of contextual factors shaping the policy processes.

Lao People's Democratic Republic (Lao PDR) is a low-income country in Southeast Asia. The GDP is 428 USD per capita [23], and the health indicators are among the lowest in the region. Lao PDR is an authoritarian, one-party state guided by neo-liberalism in the economic sphere and Marxist-Leninist thought in the political sphere, both of which shape the policymaking processes. Economic liberalisation, initiated in 1986, had a strong impact on changing policies in many sectors, including health. Private pharmacies, for example, started to mushroom and drugs became widely available. The problem became that people largely self-medicated and that fake and substandard drugs were frequent, thereby increasing the risk of continued ill-health and drug resistance [24].

In 1993, the Lao government endorsed a National Drug Policy (NDP) in order to improve the situation in the health sector [24]. The five most populous provinces were selected as pilot provinces for special implementation measures of the NDP and supported by the Swedish International Development Cooperation Agency (Sida) as part of bilateral health collaboration [21]. While the policy as such was a success in terms of putting pharmaceuticals on the political agenda, its implementation was too slow. To make the implementation of the NDP more effective, HSR was introduced to strengthen the national capacity and to improve knowledge among health officials about the impacts and problems of NDP implementation. The first experiences with the HSR projects turned out to be positive and some of the results served as a basis for the NDP revision in 2001 [21]. Another five projects were initiated to continue capacity development, some with the same participants and some with new ones (the projects are presented in Table 1 and 2). The HSR training programmes were conducted during 1998–2000 and 2001–2003, with 25–30 participants for each period. The programme included theoretical parts and implementation of five projects per period. The project participants came from the pilot provinces and from the central level (mainly Ministry of Health, but also from the National Radio and the Health Technology School). Several participants were in positions to influence policy, creating a unique group in terms of geographical representation and first-hand knowledge about the projects and their usefulness for policymaking. This article draws on this knowledge when analysing the link between research and policymaking in the Lao context.

**Table 1:** The first round of HSR projects

1.	*Can health messages reduce irrational use of antibiotics? *(study in one pilot province) High percentage of self-medication of antibiotics among people who know very little about side-effects and risk factors related to the drugs.
2.	*Use of traditional medicine in Champassack Province*. (study in one pilot province) The number of people who had used traditional medicine was high (77%). However, there was lack of knowledge on how to use it properly in many situations. [41]
3.	*Knowledge, attitudes and perception about quality of drugs*. (study in one pilot province) The majority of drug sellers, customers, and villagers were not concerned about quality of drugs, and knew very little about quality issues. [42]
4.	*Effectiveness of "feedback" for improving quality of treatment based on Standard Treatment Guidelines: A randomised trial at provincial hospitals*. (study in all five pilot provinces and three non-pilot provinces) Case management of patients with malaria and diarrhoea were improved in provincial hospitals after an educational intervention using feedback of treatment indicators. [43]
5.	*Towards an effective National Drug Policy implementation*. (study in one pilot province and one non-pilot province) The implementation of the NDP programme improved the knowledge of the health administrators and the availability, quality and rational use of drugs in pilot compared to control province. [44]
6.	*Regulation of private pharmacies in Savannakhet Province *(study in one pilot province) The quality of private pharmacy practice and drugs was low with statistically significant improvements after the regulatory intervention. [45]

**Table 2:** The second round of HSR projects

7.	*Antimicrobial Self-medication for Reproductive Tract Infections in Two Provinces in Lao PDR*. (study in two pilot provinces) More than three-quarter of respondents self-medicating for RTI/STI with antimicrobials used inappropriate drugs bought from private pharmacies. RTI/STI management should be improved, including health promotion through interventions at community level and to health providers, including private drug sellers. [46]
8.	*Improving Performance of Drug Therapeutic Committees in Lao PDR*. (study in eight provinces) DTC performance improved significantly with an educational feedback intervention. The Lao DTCs need to be restructured by recruiting DTC task forces with available time dedicated to DTC activities. [47]
9.	*Improving Good Pharmacy Practice (GPP) at Private Pharmacies in Rural Areas of Vientiane Province*. (study in one pilot province) The drug sellers had limited knowledge on information of drug use provided to customers, especially on antibiotics. There was a high percentage of self-prescribing, also for antibiotics. Positive was that all drug sellers preferred advising patients to use tablets instead of injections, and that almost all customers received information on drug use seven out of ten could repeat. Written information was lacking.
10.	*Accessibility of essential drugs in remote areas in Lao PDR*. (study in one pilot and one non-pilot province) Low availability of essential drugs and inadequate management system for village revolving drug funds (RDFs). Only one-fourth to one-third of households utilised village RDF. Performance and sustainability of RDFs are still challenging.
11.	*Developing Tools for Information on Population Drug Use in Lao PDR*. A module for information on drug use was developed and recommended to be used in the next National Health Survey.

## Methods

We used a case study design [25] focusing on the role of HSR when using in-depth interviews, group discussions and a self-administered semi-structured questionnaire with health officials from provincial, as well as central level, participating as researchers in the HSR projects or in research dissemination. Lao PDR is an arduous research environment concerning access to data, and a certain degree of flexibility is needed in the research design. The various techniques for data collection were chosen accordingly, in order to capture both the perceptions among a larger group of people who would otherwise be difficult to access, and the more elaborate opinions of a select group of people familiar with the HSR programme. We found this strategy fruitful in order to strengthen the validity of the results.

The combination of authors with different backgrounds has enriched the research by producing a study that includes both a health system and a social science approach, regarding both theoretical and empirical aspects. The social scientists (CJ and KJ) conducted 28 interviews with the participants from the HSR projects in August 2003 in order to gain in-depth knowledge about the role of the HSR projects in policymaking. The interviewees had medical or pharmaceutical training (except one journalist), and the majority were from the Vientiane area working at the central Ministry of Health, in regional or central hospitals or in provincial health offices. Eight of the interviewees had participated in two HSR projects, nine in only the first round of projects, and eleven only in the second round. At least two participants from each project were interviewed. The interview questions concerned the following issues: the individual's role in and experience of the HSR project; the usefulness of HSR findings and HSR methodology applied to daily work; and the future use of the HSR projects, including dissemination of results to policymakers and the public. Most of the interviews were carried out in English, otherwise a translator accompanied the interviewers (all but two interviews were conducted by two interviewers). Each interview took around one hour, and anonymity was guaranteed.

Seven participants from the provinces were interviewed in September 2003 in a semi-structured group interview (by RW and SK), as they could not take part in the first round of interviews.

In order to increase the validity and to avoid that the political scientists had misinterpreted the respondents' answers because of their lack of knowledge about the HSR projects, GT and RW conducted a group discussion in September 2003, in English, based on a summary of the conducted interview answers with five of the 28 interviewees who spoke good English and had been particularly active during the HSR projects and courses. They also had a wide knowledge of the projects and the policymaking processes at the Ministry of Health.

A questionnaire (in Lao language) was distributed at a dissemination seminar in September 2003 for 120 specially invited health officials from the central and provincial levels, together with 28 of the project participants. Out of the 56 respondents (57 percent were men), 52 percent were medical doctors, 2 percent nurses, 25 percent pharmacists, and 20 percent other. The majority were heads or (deputy) directors of departments, divisions or hospitals, while 15 percent were technical staff. Nearly three out of four (73%) came from the five pilot provinces and 14 percent from non-pilot provinces, while 12 percent were from the central level. The questionnaire was to a large extent identical to a survey conducted on health officials at a National Drug Conference in 2001 and comprised both closed and open-ended questions [1]. This has made it possible to identify attitude changes over time, even if the participants were not all the same and the HSR projects being discussed were different. The questionnaire consisted of 11 questions (seven of them open-ended) relating to profession and affiliation, knowledge and usefulness of operational research in general and presented at the seminar, and the quality of dissemination of research results. In all, 56 participants filled in the questionnaire anonymously. The answers were later translated into English.

## Results

### The interviews

In the interview results, a clear distinction can be found between two different effects of the HSR projects: capacity development as a result of participation in the projects and political action on the basis of research findings.

#### Capacity development

All interview respondents assured that they had learned a number of useful things from participation in the programme. The vast majority had no prior experience of HSR or the methods used and only a few had conducted clinical research. The training thus provided new knowledge about methodology, data collection and data analysis, as well as the drafting of project proposals and final reports. Teamwork and the management of research teams were other things learned in the process. Exposure to the English language also proved beneficial to participants. The interviewees indicated some factors facilitating the capacity development process, but also pointed to a number of obstacles.

Network building was a facilitating factor. As several projects involved fieldwork at provincial and community levels, they contributed to contacts between central and peripheral actors and to the emergence of informal networks among the participants. The fieldwork also gave new, positive experiences as some project participants had never had any close encounters at the grassroots level and had previously only received second hand information about local conditions.

The major obstacles for learning about research methodology and about how to conduct research were lack of time, too few participants, and language barriers. First, and most important, time spent in training and research was too short to really learn, understand and absorb all the new knowledge. As one respondent put it, "participants did not become good researchers after participating in one project."

Participation among team members in each project was uneven. The Principal Investigator (PI) carried a heavy burden, especially in the more difficult analytical tasks, usually drafting both the project proposal and the final report. Moreover, the PIs had to spend a lot of their time translating and explaining to participants with little knowledge of English. Also, as some respondents pointed out, continuity is needed to uphold the achieved capacity development. Participants need to be involved in new research beyond this specific HSR experience.

Most interviewees professed their willingness to participate in research in the future, if the opportunity should arise. Yet a few of the medical doctors, working as clinicians, admitted that they would prefer clinical research to health system research. They stated that HSR was not really their own field and that they felt uncertain at the outset whether they could really carry out the kind of research requested. Notwithstanding the expressed usefulness of their participation in the HSR training and research, they would feel more comfortable in the future with clinical research. Yet others stated that they had learned to appreciate HSR more than clinical research because of its perceived importance in the Lao context where this kind of research is lacking.

#### Turning findings into political action

Whereas respondents frequently lamented the lack of direct action on the basis of HSR findings, they also pointed to positive indirect effects and areas where practices have changed as a result of the HSR programme. For example, drug information and licensing have improved, indicators of pharmacy practice developed within the programme have helped in continued monitoring, and a new, improved edition of the Standard Treatment Guidelines has been published. For other actions to follow more time was said to be needed and some respondents spoke of five to ten years. While mentioning but a few facilitating factors for turning findings into action, our interviewees identified several barriers along that road.

The main facilitating factors were "researchers-cum-administrators" and internationalisation. Some of those participating in the HSR projects have since assumed high administrative positions, facilitating the dissemination of research results to the policymakers through personal contacts. Internationalisation puts pressure on Lao PDR to keep up its research capacity and to turn findings into action. One example is the need to provide input regularly to the World Health Report by WHO. International contacts also make research funds more accessible.

The obstacles mentioned were lack of money, attitudes towards research, the lack of ownership, the lack of early involvement by policymakers, the difficulties of research communication and the lack of tradition in lobbying. The absence of action was most commonly attributed to the lack of indigenous funds for health system reform and a lack of HSR culture and recognition by policymakers for its need. Respondents typically expressed their worry about the discontinuance of Sida funds (which have financed the HSR projects). To follow up on the HSR projects, funding from other donors was perceived to be needed.

The interviews revealed cultural obstacles, as well. Several respondents emphasised current attitudes to research in Lao PDR, among medical staff and policymakers alike. Research, in short, does not have the positive connotations that it has in most high-income countries. Medical staff is accustomed to working by routine and tends to be suspicious of research. For example, some of the persons selected for interviews declined to be interviewed, ostensibly because it was not "their area". There is no real appreciation of research in Lao PDR, no tradition among medical staff to follow international professional journals, and limited incentives to publish internationally, even if it is important to publish in Lao. Only those who want a master's degree or PhD and/or plan to study abroad are interested in publications, as it can be useful for their career. However, the introduction of professorships and associate professorships has started to change this, according to the participants of the group discussion.

Among some policymakers, according to our interviewees, research was seen as expensive and as a waste of scarce resources. Besides, policymakers claim to know the situation in their own country, and are not persuaded by "studies of just one province." They do not always see the usefulness of research findings, and sometimes deny or do not want to acknowledge findings that may be inconvenient either for their ideology or their career. Provincial policymakers were seen as having less understanding of research than those working at the central level. However, some of the interviewees stated that policymakers have a good understanding of research, but that they in one way or the other are restrained to take action.

Some respondents pointed to the need to get policymakers onboard before launching research projects in Lao PDR. Whereas policymakers were indeed consulted in planning the HSR, they did not have much time and never became really involved. One suggestion was to include policymakers early, already at the point of brainstorming about possible topics for HSR.

The participating researchers also need improved skills in research communication, according to our interviewees. There is a need to summarise the most important findings from the programme and to present them in different, appropriate forms to the general public, media and policymakers. One of the respondents, who had participated in training abroad on how to formulate policy briefs, suggested that future research projects should include training on research communication to both colleagues and policymakers. Related to insufficient communication skills is the lack of any lobbying tradition in Lao political culture. "I can only show data," as one respondent put it, "then it depends on policymakers to act upon them." Even if several interviewees talked about the need to spend more time and efforts to persuade, and explain to, policymakers, it was obvious that very few saw this as their responsibility.

### Group interview and group discussion

The same questions, as in the individual interviews, were asked during the group interview with participants from the provinces. The group interview confirmed the previous findings. However, these interviewees were, in general, more positive towards the usefulness of the HSR projects, and several of the group members stated that they felt better equipped after the training, and that they used their new knowledge in their daily work.

Members of the group discussion having especially active participants agreed overall with the conclusions presented in the interview summary, but for some minor issues of disagreement. However, the issues concerned individual points of view rather than a general view of all the interviewees, and they were almost exclusively results of ambiguous phrasing in the summary. Hence, after crosschecking with the interview notes, the summary was reworded accordingly in order to avoid misunderstandings.

### The questionnaire

Practically all respondents (91%) thought that operational research supports evidence-informed decision-making. Almost two thirds (62%) had heard about operational research previously, through meetings and workshops, overseas and from Lao research teams, or through information from Ministry of Health and at hospitals and pharmacies. Written material had also been a source of information. The majority found the information about research at the conference useful or very useful (95%). The reasons were many, but a common reply was the need for reality-based information. More than two-thirds (71%) stated that they use research results in their daily work (primarily research results about the use of drugs). However, almost all (93%) said they want to use research results in their daily work in the future.

From the open-ended part of the questionnaire we found that the most important research results derived from the HSR projects identified the problem of self-medication of antibiotics and the problem of reaching (poor) people in the remote areas with information and drugs.

Suggestions for improving the presentation and communication of research results included dissemination of research summaries to provinces, as well as to policymakers at the central level, along with instructions on how to use the results practically. The results should be published in bulletins, newspapers, television and radio. The information could also be useful for other sectors, both domestically and internationally. Research results should be presented regularly.

Despite the very positive attitudes, in general, towards research and a wide-ranging call for the dissemination of findings from the conference, some respondents expressed fear over how to translate these findings into practice. One wrote: "I know all the findings, but I worry about how to utilise them properly".

Many of the respondents stated that there has been a general change of attitude during the last two years towards the use of research results for decision-making in the health sector, concerning especially the rational use of drugs for treatment, drug therapeutic committees and standard treatment guidelines. Because increasing numbers of people are involved in research projects in Lao PDR, the leaders are beginning to see the role of research. However, even if research can be used for decision-making, there might be a tendency to ignore information if it contradicts current policy or implementation.

## Discussion

We found that interviewees and questionnaire respondents were aware of the barriers to getting research into policy and practice. Secondly, while some were optimistic of change others were more pessimistic. The optimists claimed that there had been a change of attitudes among policymakers in the last two years because of the exposure to HSR, while the pessimists did not expect any real changes until years from now, indicating the ambivalent role of research in the Lao context. Thirdly, the major barriers mentioned for feeding research results into policy and practice were an inability to influence the policy process or to get policymakers and practitioners interested in research results. Another barrier was the lack of continuous capacity development and high-quality research, both of which are related to funding and international support. Finally, many of the interviewees and questionnaire respondents also pointed out that communication between those conducting research and policymakers must be improved.

The identified barriers are practically identical to what has been identified by others focusing on the perceptions of policymakers [2,6,26], and correspond to what has been described as the four conceptual categories of political context, evidence, links and external influences [13,27,28]. By highlighting these broad categories in relation to our major findings, a more nuanced picture emerges.

Understanding our findings within the category of political context dictates that the people, institutions and processes must all be placed specifically within the Lao policymaking context [26]. According to Court and Young [29], the most important factor affecting the uptake of research into policy is the political institutional context. In Lao PDR power is concentrated to a small elite closely related to the communist party, and the lack of transparency makes the policy process largely opaque for an outsider [21], even if the health sector is more open than many other sectors. Consensus is important and decisions have by tradition been guided more by political concerns and ideology than by research evidence [30]. The use of pilot projects provides practice, meaning that many measures are implemented before becoming an official policy; and depending on how results of the projects are received, the project may or may not be replicated at a national level. The policy process takes time and one cannot expect rapid changes, as expressed by some of our interviewees. The system also limits room for manoeuvre for health officials to introduce new ideas. The social structure is hierarchical, and the habit of waiting for instructions from above in order to avoid criticism does not encourage individual initiatives [31]. This can explain why few of the interviewees saw the dissemination of research results as their responsibility, and why it is more important with "know-who" than "know-how" in influencing policy. Also, a low-income country such as Lao PDR has many battles to fight, and research does not automatically top the agenda, including HSR. In general, many policymakers are ignorant of policy-relevant research [14], and this is true for Lao PDR, as well. Research "of just one province" may not be perceived as enough, especially if the policymakers do not know how to use the results, or if the results contradict the interests of the policymaker. Even if the research results reach the implementers at grassroots level, these "street-level bureaucrats" face difficulties when they have to translate research into practice [32]. The lack of resources, instructions about what to do and resistance against change can create almost insurmountable obstacles for reforms [21]. Our material shows that the dissemination of research results requires attitude change among medical staff as well as policymakers. Whereas some respondents saw some development in the direction of more positive attitudes to research, they admitted that this is only the beginning of a long-term process. Another aggravating factor was that many in the medical profession do not see evidence-based research to be in their own interest.

The lack of solid evidence, or high-quality research, creates problems for feeding research into policy [13]. Evidence, as categorical concept, concerns not only the type and quality of research, but also how the research is communicated. Communication is related to capacity development. The need for more HSR together with more training, i.e. continuous capacity development, was widely acknowledged by some of our interviewees. Problems in being able to implement research findings into policy can be derived from a lack of higher education, literature, research methods and tools, such as computer software. Still, most LMIC spend far too little on research, Lao PDR included [33]. Moreover, weak institutional capacity to absorb (external) funds and too few trained researchers to support mechanisms to sustain capacity built up through the projects hamper the catering of research to governmental policy development. The HSR projects in Lao PDR have been instrumental for capacity development of individuals, and ideally, participants in the various projects will transmit this new knowledge to widening circles of colleagues. A number of participants in the HSR programme have since become involved in various teaching and training programmes, where they transmit to others their methodological knowledge about how to conduct research and use HSR projects as educational examples or cases. But the critical mass of skilled researchers needed for affecting policymaking at a larger scale is still not yet in place, even if lessons from the HSR projects have been incorporated into curriculum and a growing number of health officials have been trained and have earned master or doctoral degrees abroad. For many participants of the HSR projects, it was the first time they conducted research, several of them only part-time or during free time. They had been appointed to participate in the HSR programme, which probably can explain some of the participants' reluctance to HSR. Another challenge was the introduction of qualitative research [34] together with analytical, critical evaluation. At the same time, our material supports past research [35] that the more educated the HSR participants were, the better they could make use of the HSR and produce good research. In fact, seven articles have thus far been published in international peer-reviewed journals (see Table 1 and 2) of the eleven HSR projects that have been initiated. The results have been used directly by practitioners or more indirectly in policymaking, indicating that research still has mostly an "enlightening function" in the policy process. This can be regarded as reasonable, as it is relatively uncommon that research can claim direct effects on policy change [26,36]. It should be noted that there has been a lack of knowledge about the health care system in general, which probably contributes to less interest in finding solutions. The HSR projects show that relatively small efforts can lead to substantial change in the perception of the usefulness of research.

According to our final major finding, the communication between the Lao researchers and policymakers could be improved, if links between them were strengthened. Links, such as networks or media, trusted "dissemination agents" or "translators", between researchers and policymakers are central in communicating research results [17], both at the national and international level. At the national level, Lao PDR has an advantage in the size of the policy community in the health care sector, which is relatively small, and in the blurred line between "researchers" and administrators. This together with the design of the HSR projects, where health officials from all over the country participated, has resulted in relatively widely disseminated HSR results. The design of the HSR projects has also meant that a network between members from the central to the provincial level has developed, which will be useful far beyond the specific HSR projects. Members of this network may serve as brokers to introduce new ideas into policymaking circles [37]. It is interesting to note, however, that national networks are inadequate in an increasingly globalised world. International networks will increase in importance, as policy processes become increasingly global [27]. Media has played a positive role by publishing research results, especially via radio broadcasting, but in Lao PDR it thus far plays no role in setting the research agenda. There is a lack of lobbying traditions, interest groups such as consumers or patient organisations, and public debate about health care that could push for the use of research in policymaking.

External influences, such as bilateral donor policies, were perceived as something both positive and negative by our interviewees. International contacts are crucial to develop good research, but at the same time the dependence on external funding is a cause of worry. In aid-dependent countries, such as Lao PDR, donors set a large part of the research agenda [38]. A donor can create independence and serve as a link to the latest international thinking, but it can also create research agendas with little relevance for the country's policy context [13,14,27,39], the latter with consequences for ownership and legitimacy as well as the question of who is going to be responsible for getting research into policy and practice [11,29]. According to Maxwell and Stone [14], policy impact is highest when research is carried out through collaboration with international research institutes over a longer time-period. This can be compared with the support for implementation of the NDP that has lasted for over a decade, and in addition to research training and projects, has included seminars, conferences, study tours, and discussions with high-level policymakers. It is only recently that real changes concerning the attitudes and use of HSR can be noted, according to our interviewees, confirming the need for long-term support. Furthermore, according to some of our interviewees, policymakers need to be involved in research with a vision in order to get ownership and not be too dependent on donors. This reflects the problem of sustainability, and how this affects the commitment to research. At present it may be necessary that donors play a major role in setting the research agenda in resource scarce countries such as Lao PDR. In this case, the donor (Sida) and its consultant agency were crucial for respectively financing and initiating, conducting and disseminating the HSR projects and the results. The introduction of HSR has lead to more appreciation of research, which in turn may lead to a higher degree of Lao ownership and commitment to research in the long run. At the same time it is important to be aware of contextual particularities when using external support, and it is essential to develop research and capacity development methodologies that are adapted to the local context.

## Limitations of the study

This study has limitations that are to some extent also its strengths. The involvement of both medical doctors and social scientists has enriched the study by providing different perspectives on the subject matter. However, a multidisciplinary approach also includes negotiations about how to design and conduct a study. In this case, the result is a study about HSR elaborating on political aspects and structural barriers in policymaking through in-depth interviews at the expense of more quantitative methods of analysis that could have highlighted other issues. At the same time, the way the data was collected and crosschecked contributed to a comprehensive picture of the difficulties in capacity development for feeding research results into policy and practice. Another limitation is that we only got the view of HSR participants and not of policymakers, which could have created a more nuanced picture of the impact of HSR on policy and the role of HSR participants in research communication. However, this study is explorative in character and should be valued as an innovative approach to investigate how to implement research into policy and practice by its focus on perceptions and capacity development processes among HSR participants.

## Conclusion

As shown in this study, the participants of the research projects had knowledge about the problems of getting research into policy and practice, and yet the majority were motivated to participate in further research projects if the opportunity arose. Some research results have been implemented, such as improved drug information, indicators of pharmacy practice and an improved edition of the Standard Treatment Guidelines, which shows that research evidence in some cases has been both convincing and has provided a practical solution, supporting the idea that even smaller HSR projects may have an impact on policy. For several of the participants of the HSR projects there are more HSR underway through a new project on ill-health and poverty, where the political commitment to poverty reduction and the fulfilment of Millennium Development Goals may help to create a new "window of opportunity" for using research in policymaking.

This implies that there is a foundation for evidence-informed policymaking. However, there are substantial barriers to overcome if research is going to have a more profound impact on policymaking. As it is now, the lack of national institutional arrangements for continuous research capacity development and follow-up research, in combination with a lack of long-term international support, prevent the necessary critical mass for making research a natural ingredient in policymaking. Further capacity development is needed on all fronts, but must be contextualised and adapted to fit the political and socio-economic situation in Lao PDR, as this study has shown [cf. [40]]. Resources are scarce in Lao PDR and politics is a top-down endeavour, hence it is important that earmarked resources for research are available and that research policy is well anchored among the leadership if change is going to take place.

## Competing interests

GT and RW have been coordinators of the support for implementation of the NDP programme and of the HSR projects. Otherwise the authors have no competing interest.

## Authors' contributions

KJ is the main author. The other authors have written parts of the text and/or commented on the text. KJ and CJ conducted and analysed the interviews, RW and GT conducted the group discussion, RW and SK conducted the group interview. SK also served as a translator during a number of the interviews, and worked with the translation of the questionnaire responses. All authors have read and approved the final version of the manuscript.
